# Identification of Hub Genes Associated With Non-alcoholic Steatohepatitis Using Integrated Bioinformatics Analysis

**DOI:** 10.3389/fgene.2022.872518

**Published:** 2022-04-26

**Authors:** Qingnan Meng, Xiaoying Li, Xuelian Xiong

**Affiliations:** The Key Laboratory of Metabolism and Molecular Medicine of the Ministry of Education, Department of Endocrinology and Metabolism, Zhongshan Hospital, Fudan University, Shanghai, China

**Keywords:** non-alcoholic steatohepatitis, differential gene expression analysis, weighted gene co-expression network analysis, protein-protein interaction, hub genes

## Abstract

**Background and aims:** As a major cause of liver disease worldwide, non-alcoholic fatty liver disease (NAFLD) comprises non-alcoholic fatty liver (NAFL) and non-alcoholic steatohepatitis (NASH). Due to the high prevalence and poor prognosis of NASH, it is critical to understand its mechanisms. However, the etiology and mechanisms remain largely unknown. In addition, the gold standard for the diagnosis of NASH is liver biopsy, which is an invasive procedure. Therefore, there is a pressing need to develop noninvasive tests for NASH diagnosis. The goal of the study is to discover key genes involved in NASH development and investigate their value as noninvasive biomarkers.

**Methods:** The Gene Expression Omnibus (GEO) database was used to obtain two datasets encompassing NASH patients and healthy controls. We used weighted gene co-expression network analysis (WGCNA) and differential expression analysis in order to investigate the association between gene sets and clinical features, as well as to discover co-expression modules. A protein-protein interaction (PPI) network was created to extract hub genes. The results were validated using another publicly available dataset and mice treated with a high-fat diet (HFD) and carbon tetrachloride (CCl4).

**Results:** A total of 24 differentially co-expressed genes were selected by WGCNA and differential expression analysis. KEGG analysis indicated most of them were enriched in the focal adhesion pathway. GO analysis showed these genes were mainly enriched in circadian rhythm, aging, angiogenesis and response to drug (biological process), endoplasmic reticulum lumen (cellular component), and protein binding (molecular function). As a result, eight genes (JUN, SERPINE1, GINS2, TYMS, HMMR, IGFBP2, BIRC3, TNFRSF12A) were identified as hub genes. Finally, three genes were found significantly changed in both the validation dataset and the mouse model.

**Conclusion:** Our research discovered genes that have the potential to mediate the process of NASH and might be useful diagnostic biomarkers for the disorder.

## Introduction

Accompanied by the global increase in obesity, non-alcoholic fatty liver disease (NAFLD) is currently a primary cause of liver disease worldwide (Massoud and Charlton, 2018). Non-alcoholic steatohepatitis (NASH) is one of the forms of NAFLD, and is characterized by lobular inflammatory infiltrates, hepatocyte ballooning, and cell death. It may progress to hepatic fibrosis, cirrhosis, and ultimately hepatocellular carcinoma (HCC). NASH is currently the leading cause of liver-related mortality and the second-leading cause of liver transplantation in many western countries (Younossi, 2019). NASH has a close correlation to risk factors such as overweight, type 2 diabetes, dyslipidemia, hypertension, inflammation and is considered the hepatic manifestation of metabolic syndrome (Chalasani et al., 2018; Sanyal, 2019).

Previous investigations have provided deeper insights into the evolution of NASH. One of the widely accepted theories is that the overloaded metabolic substrates lead to liver injury in NASH (Friedman et al., 2018). However, the exact mechanisms underlying NASH are still not fully explored. In addition, although liver biopsy is currently the gold standard for NASH diagnosis, it has a number of disadvantages, such as sampling error, cost, and risks of complications. Therefore, there is a demand to discover non-invasive diagnostic biomarkers for NASH (Sumida et al., 2014).

Bioinformatics has been increasingly used to analyze the principles of diseases and detect disease-specific biomarkers (Can, 2014). Weighted gene co-expression network analysis (WGCNA) is a method to identify gene function and the correlation between genes and clinical traits (Langfelder and Horvath, 2008). It can find gene modules that are closely related to specific diseases by analyzing the relationship between genome and clinical information (Zhang and Horvath, 2005), providing insights into functions of co-expression genes and identifying key genes in diseases (Saris et al., 2009; Yang et al., 2014). Differentially expressed gene (DEG) analysis is another approach to studying mechanisms of diseases by discovering quantitative differences in expression levels between groups (Segundo-Val and Sanz-Lozano, 2016).

In this study, we obtained the mRNA expression data from the Gene Expression Omnibus (GEO) database. DEG analysis was conducted to explore the correlation between genes and clinical traits, and WGCNA was performed to discover the co-expression modules. Then the overlapping genes in the intersection of DEG lists and co-expression modules were selected and further characterized through GO and KEGG analysis. An overlapping gene-based protein-protein interaction (PPI) network was used to identify hub genes. Finally, the hub genes were validated by another dataset from GEO as well as a mouse model of NASH. This study offered a theoretical foundation for further understanding the processes of NASH by identifying differentially co-expressed genes, which may also be promising biomarkers for the existence of NASH.

## Materials and Methods

### Workflow

**FIGURE 1:**
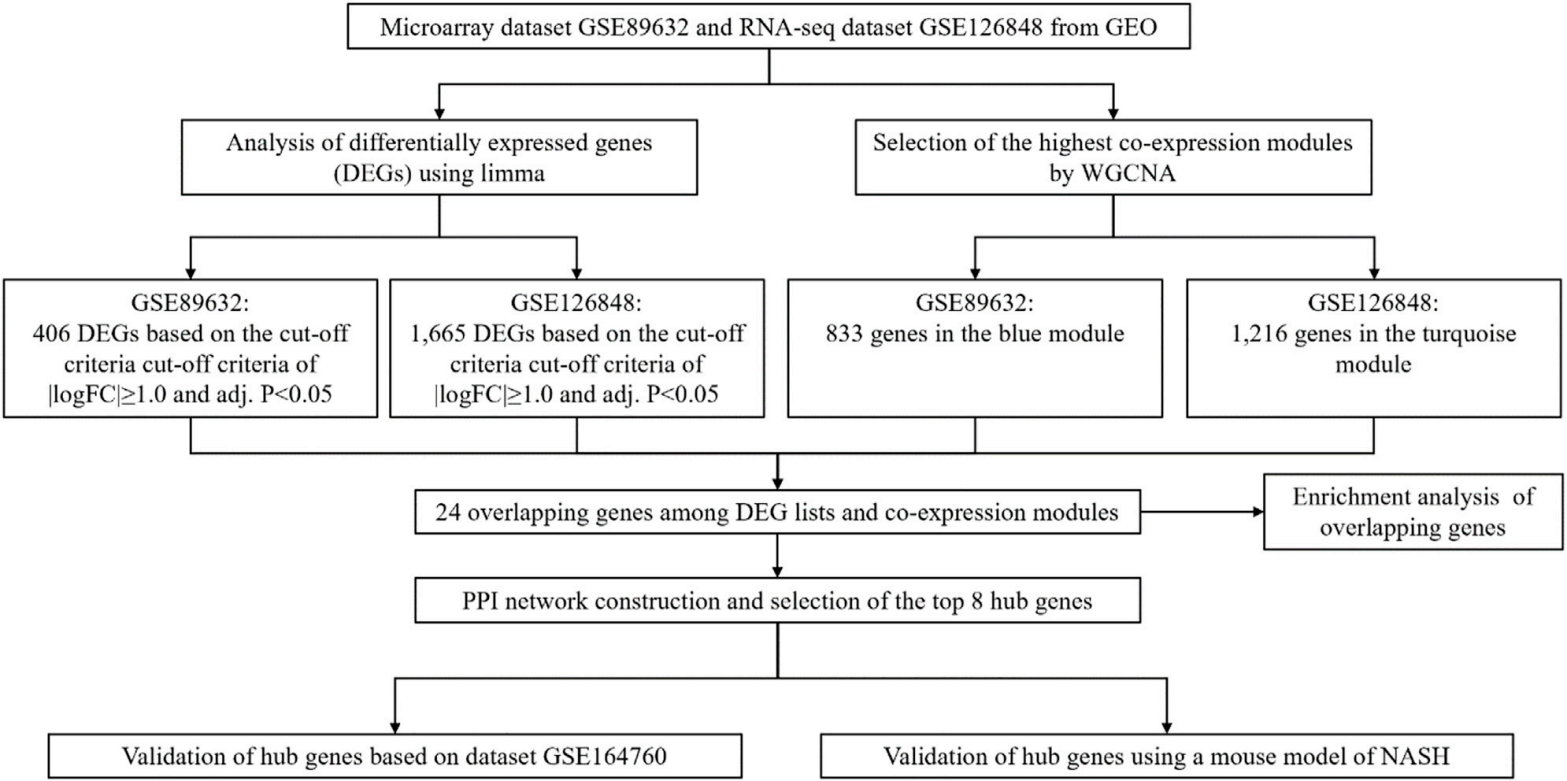
Workflow of this study.

Figure 1 illustrates the workflow of the study including data extraction, processing, analysis, and verification.

### Data Collection and Processing

Datasets GSE89632 and GSE126848 were downloaded from GEO (http://www.ncbi.nlm.nih.gov/geo) for WGCNA and DEG analysis. GSE89632 included liver tissues from 19 patients with NASH and 24 healthy controls (HC) and was studied with the GPL14951 platform Illumina HumanHT-12 WG-DASL V4.0 R2 expression beadchip. GSE126848 was comprised of liver tissues from 16 NASH patients and 26 HC and was studied using the GPL18573 platform Illumina NextSeq 500. Probes were translated into gene symbols, and repeated probes for the same gene were eliminated by calculating the median expression value of all associated probes. Details of the datasets are shown in Table 1. Consequently, 29,377 and 19,786 genes from GSE89632 and GSE126848, respectively, were chosen for further investigation.

**TABLE 1:** Characteristics of the included GEO datasets.

	HC	NASH	Experiment Type	Tissue
GSE89632	24	19	Expression profiling by array	Liver
GSE126848	26	16	Expression profiling by high throughput sequencing	Liver
GSE164760	6	74	Expression profiling by array	Liver

### Weighted Gene Co-Expression Network Analysis

In this study, the *WGCNA* package in R (Langfelder and Horvath, 2008) was used to convert the gene expression data into gene co-expression networks to investigate the modules of highly correlated genes. Soft-thresholds for GSE89632 and GSE126848 were chosen through the function *pickSoftThreshold*. The adjacency matrix was then created by the formula: $a_{ij}={\left|S_{ij}\right|}^{\beta }$ ( $a_{ij}$ : adjacency matrix between gene I and gene J, $S_{ij}$ : similarity matrix calculated using Pearson correlation of all gene pairs, β : soft power). Then we transformed the adjacency matrix into a topological overlap matrix (TOM) and the corresponding dissimilarity matrix (1-TOM). After this, the related genes were classified into various co-expression modules using a hierarchical clustering dendrogram of the 1-TOM matrix. To identify the module-trait associations, the clinical traits of samples were defined as NASH and HC. As a result, modules with a high correlation coefficient were chosen for the following study as they’re possibly closely related to NASH.

### Differentially Expressed Gene Analysis and Identification of Overlapping Genes

In an attempt to find the differentially expressed genes (DEGs) between NASH and HC, the R package *limma* (Ritchie et al., 2015) was utilized to analyze GSE89632 and GSE126848. After converting the gene expression fold changes (FC) to log2 values, we identified genes with the cutoff criteria of $\left|\log_{2}FC\right|$ ≥ 1.0 and adjusted *p*-value < 0.05 as DEGs. Benjamini–Hochberg method was applied to adjust the *p*-values so as to control the false discovery rate (FDR). The DEGs of GSE89632 and GSE126848 were visualized as volcano plots using the R package *ggplot2* (Ito and Murphy, 2013).

The intersection between DEG lists and co-expression gene modules was utilized to identify overlapping genes, which was shown as a Venn diagram through the R package *VennDiagram* (Chen and Boutros, 2011).

### GO and KEGG Analysis of Overlapping Genes

For further understanding of the biological function and signaling pathways of the overlapping genes, the identified genes were analyzed by R package *clusterProfiler* (Yu et al., 2012) for gene enrichment analysis within the Gene Ontology (GO; http://www.geneontology.org/) database and pathway analysis within the Kyoto Encyclopedia of Genes and Genomes (KEGG; https://www.kegg.jp/). *p*-values of <0.05 were used to define the working threshold for statistical significance. The terms of GO enrichment analysis can be categorized into three groups: “biological process” (BP), “cellular component” (CC), and “molecular function” (MF).

### Construction of PPI Network and Identification of Hub Genes

Identification of hub genes was based on the protein-protein interaction (PPI) network construction. Through importing the overlapping genes into the Search Tool for the Retrieval of Interacting Genes (STRING) database (Franceschini et al., 2013), the interaction relationships among the proteins encoded by the overlapping genes were searched. Results were visualized as the PPI network by the Cytoscape software (Chin et al., 2014).

In a co-expression network, Maximal Clique Centrality (MCC) algorithm was used to select the hub genes. The MCC score of each node was calculated by the Cytohubba plugin of Cytoscape (Chin et al., 2014).

### Verification of Hub Genes Using a Dataset From GEO

We used dataset GSE164760 from the GEO database as the validation dataset, which contains data from 74 NASH patients and 6 HC. The expression information of the identified hub genes was extracted from the expression matrix of the validation dataset. Differences in gene expression between NASH and the HC group were analyzed using the Wilcoxon signed-rank test.

### Animal Experiments

Eight weeks old male C57BL/6 mice were purchased from the Shanghai Laboratory Animal Company. All mice were housed at 21 ± 1°C with 55 ± 10% humidity and a 12-h light/dark cycle. Mice were divided randomly into two groups, the HFD/CCl4 group and the healthy control (HC) group. For the HFD/CCl4 group, CCl4 combined with a high-fat diet (HFD; D12492, Research Diets) was used for induction of NASH. For 4 weeks, the HFD/CCl4 group was given 2 ml/kg body weight of a 10% CCl4 solution in oil intraperitoneally twice a week (Kubota et al., 2013). The HC group received only a standard normal diet without any intervention. Food consumption was monitored during the experiment by measuring the food during cage changes and at the end of the trial. The mice were sacrificed by cervical dislocation at the end of the treatments, and the liver tissues were snap-frozen with liquid nitrogen and kept at −80°C for further analysis. All animal protocols were reviewed and approved by the Animal Care and Use Committee of Zhongshan Hospital, Fudan University.

### Quantitative RT-PCR Analysis

Trizol reagent (Invitrogen) was used to extract total RNA from liver tissues and cells, and reverse transcription was performed with a PrimeScript RT Reagent Kit (TaKaRa, Tokyo, Japan) according to the manufacturer’s instructions. Quantitative real-time PCR was performed using a SYBR Green Premix Ex Taq (Takara, Japan) on Light Cycler 480 (Roche, Switzerland) and data were analyzed by the 2^−ΔΔCt^ method with GAPDH as an internal control for normalization. The primer sequences used in RT-PCR were shown in Table 2.

**TABLE 2:** Primers used for RT-PCR analysis.

Gene Symbol	Species	Forward Primer	Reverse Primer
*JUN*	Mouse	CCT​TCT​ACG​ACG​ATG​CCC​TC	GGT​TCA​AGG​TCA​TGC​TCT​GTT​T
*SERPINE1*	Mouse	TTC​AGC​CCT​TGC​TTG​CCT​C	ACA​CTT​TTA​CTC​CGA​AGT​CGG​T
*IGFBP2*	Mouse	CAG​ACG​CTA​CGC​TGC​TAT​CC	CCC​TCA​GAG​TGG​TCG​TCA​TCA
*TYMS*	Mouse	GGA​AGG​GTG​TTT​TGG​AGG​AGT	GCT​GTC​CAG​AAA​ATC​TCG​GGA

## Results

### Co-Expression Modules Identified Through WGCNA

**FIGURE 2:**
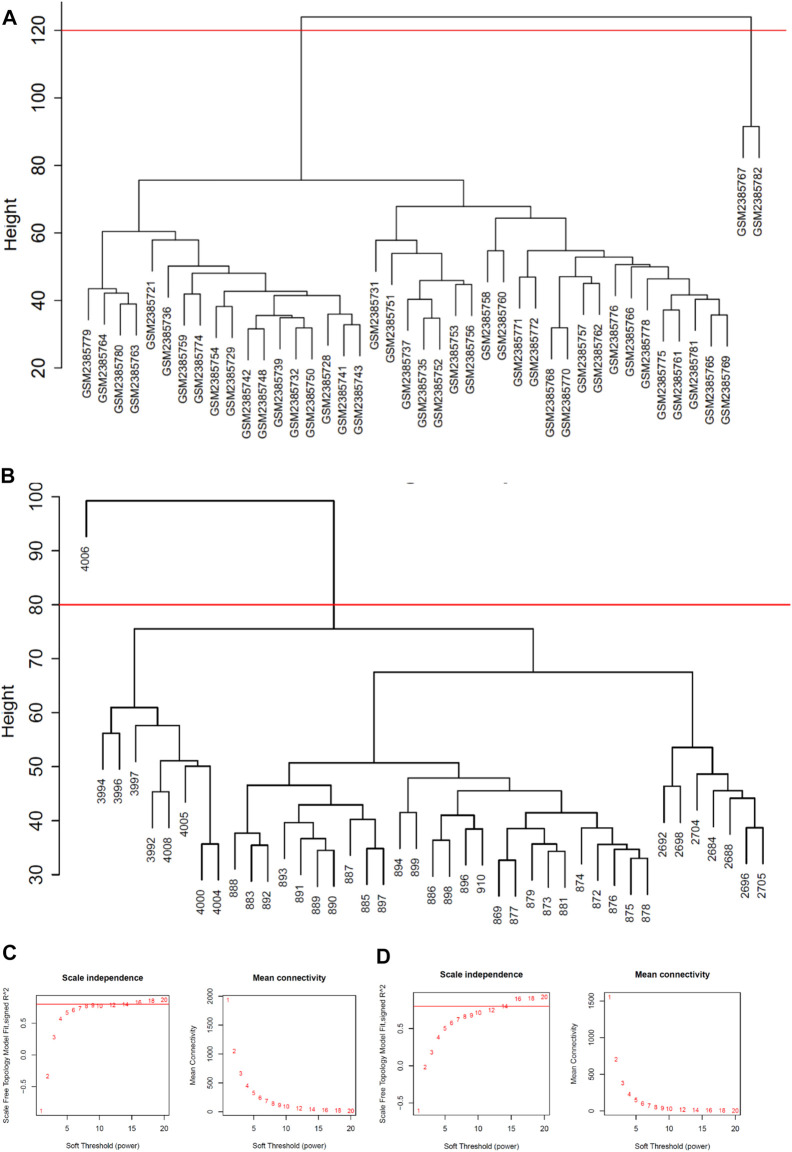
Data processing before WGCNA analysis. **(A)** Removal of outlier samples from GSE89632. **(B)** Removal of an outlier sample from GSE126848. **(C)** Soft-thresholding calculation of GSE89632; Left: scale-free fit indices using various soft-thresholding powers; Right: mean connectivity using various soft-thresholding powers. **(D)** Soft-thresholding power calculation of GSE126848; Left: scale-free fit indices using various soft-thresholding powers; Right: mean connectivity using various soft-thresholding powers.

**FIGURE 3:**
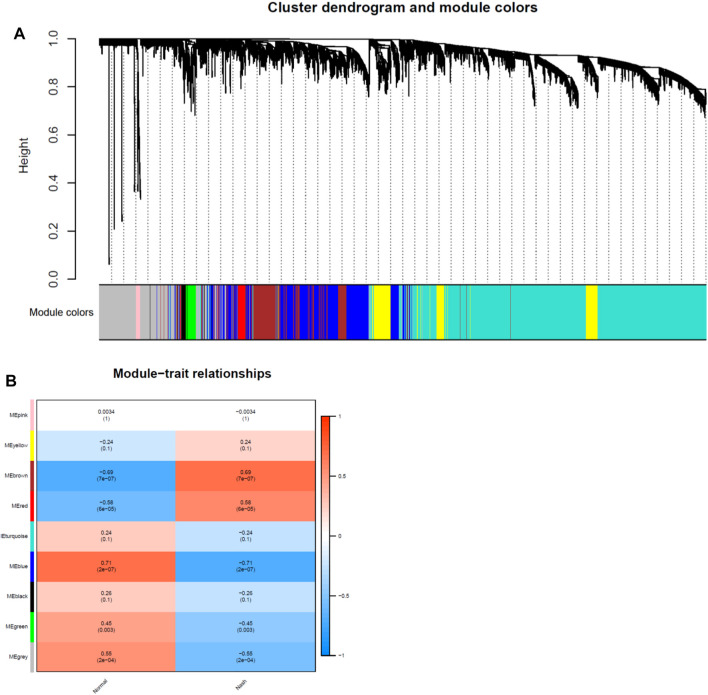
Identification of modules associated with clinical traits in GSE89632 dataset. **(A)** The Cluster dendrogram of co-expression network modules was ordered by a hierarchical clustering of genes based on the 1-TOM matrix. Each module was colored differently. **(B)** Module-trait relationships. Each row corresponds to a module and column corresponds to a clinical trait (NASH or HC). Each cell includes the corresponding correlation and *p*-value.

**FIGURE 4:**
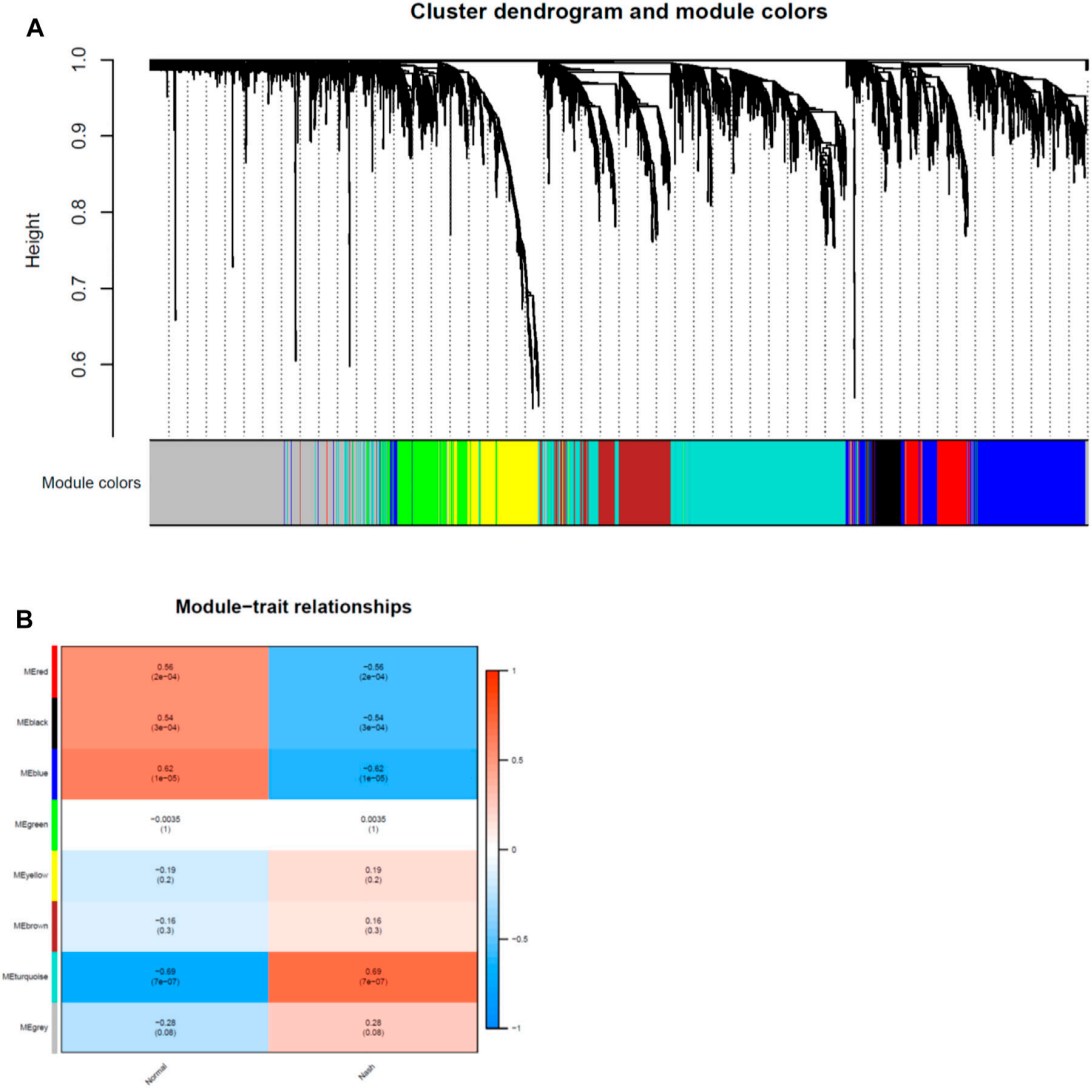
Identification of modules associated with clinical traits in GSE126848 dataset. **(A)** The Cluster dendrogram of co-expression network modules was ordered by a hierarchical clustering of genes based on the 1-TOM matrix. Each module was colored differently. **(B)** Module-trait relationships. Each row corresponds to a module and column corresponds to a clinical trait (NASH or HC). Each cell includes the corresponding correlation and *p*-value.

Network module analysis is vulnerable to outlier samples. Therefore, we built hierarchical clustering trees for all samples and removed outlier samples. As a result, we removed 2 outlier samples (GSM2385767 and GSM2385782; Figure 2A) from GSE89632 and 1 sample (sample 4006; Figure 2B) from GSE126848. Next, co-expression analysis was carried out to construct the co-expression network. In this study, the power of *β* = 16 was selected for both of the datasets as the soft thresholding powers to ensure a scale-free network (Figures 2C,D). In total, 9 modules in the GSE89632 (Figure 3A) and 8 modules in the GSE126848 (Figure 4A) were identified.

Then we created heatmaps of module-trait relationships to assess the correlation between each module and clinical traits (NASH and HC), which are shown in Figures 3B, 4B. As a result, the blue module in the GSE89632 (*P* = 2E-07) and the turquoise module in the GSE126848 (*P* = 7E-07) were the most significantly related to NASH among all of the modules, therefore the two modules were selected as clinically important modules for further analysis.

### Identification of DEGs and Overlapping Genes

**FIGURE 5:**
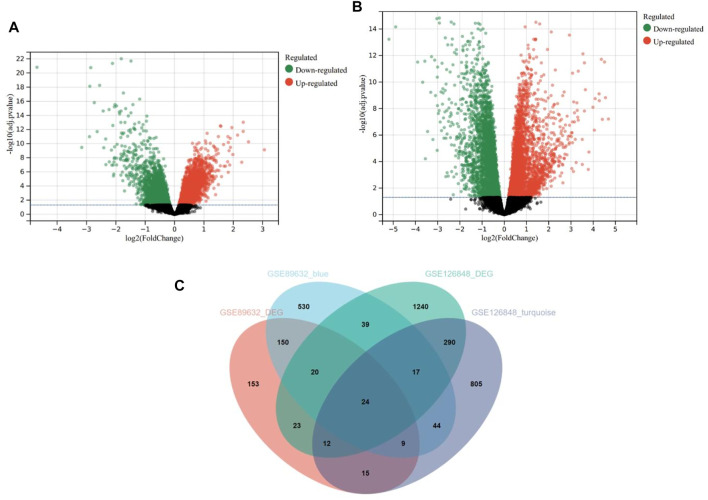
Differentially expressed genes (DEGs) in the GSE89632 and GSE126848 datasets. **(A)** Volcano plot of DEGs in the GSE89632 dataset. **(B)** Volcano plot of DEGs in the GSE126848 dataset. **(C)** The Venn diagram of genes from the DEG lists and co-expression modules.

Based on the cut-off criteria mentioned earlier, 406 DEGs in the GSE89632 (Figure 5A) and 1,665 DEGs in the GSE126848 (Figure 5B) were found dysregulated in NASH patients compared to HC. As presented in the Venn diagram (Figure 5C), 833 and 1,216 co-expression genes were identified in the blue module of GSE89632 and the turquoise module of GSE126848, respectively. As a result, 24 overlapping genes between the DEG lists and co-expression modules were extracted for identification of hub genes.

### GO and KEGG Analysis of the 24 Overlapping Genes

**FIGURE 6:**
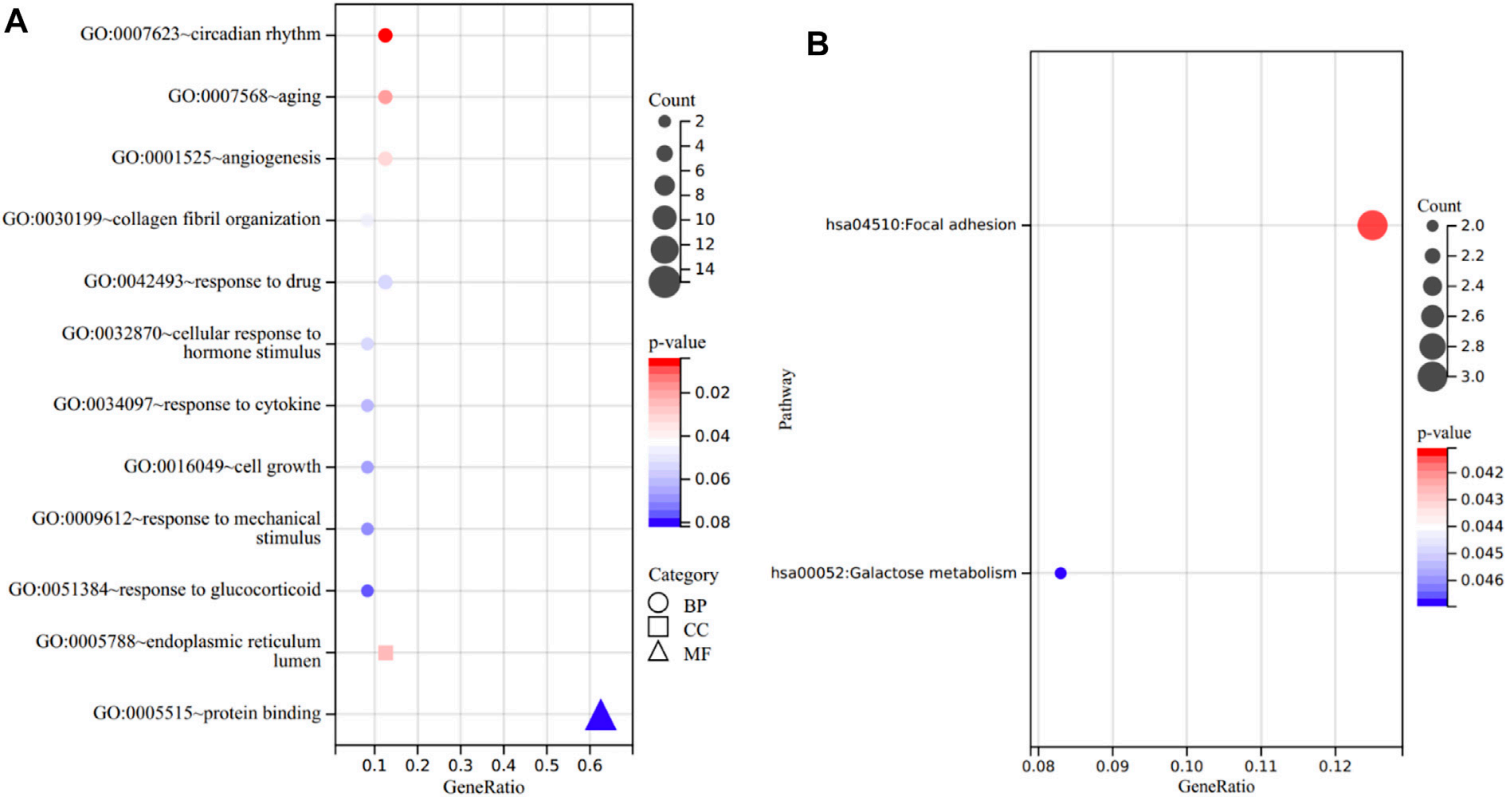
Functional annotation of the 24 overlapping genes. **(A)** The enriched terms in GO analysis. **(B)** KEGG analysis.

GO analysis consists of three sub-ontologies—biological process (BP), cellular component (CC), and molecular function (MF). The results demonstrated that, as for the BP, the genes were mainly enriched in circadian rhythm (*p* = 3.91E-03), aging (*p* = 1.78E-02), angiogenesis (*p* = 3.12E-02) and response to drug (*p* = 5.47E-02). Regarding the CC, the genes were mainly enriched in endoplasmic reticulum lumen (*p* = 2.42E-02). And as for MF, the genes were mostly enriched in protein binding (*p* = 8.18E-02) (Figure 6A).

In KEGG analysis, the enriched pathways included focal adhesion (*p* = 4.11E-02) and galactose metabolism (*p* = 4.70E-02) (Figure 6B).

### PPI Network Construction and Hub Genes Identification

**FIGURE 7:**
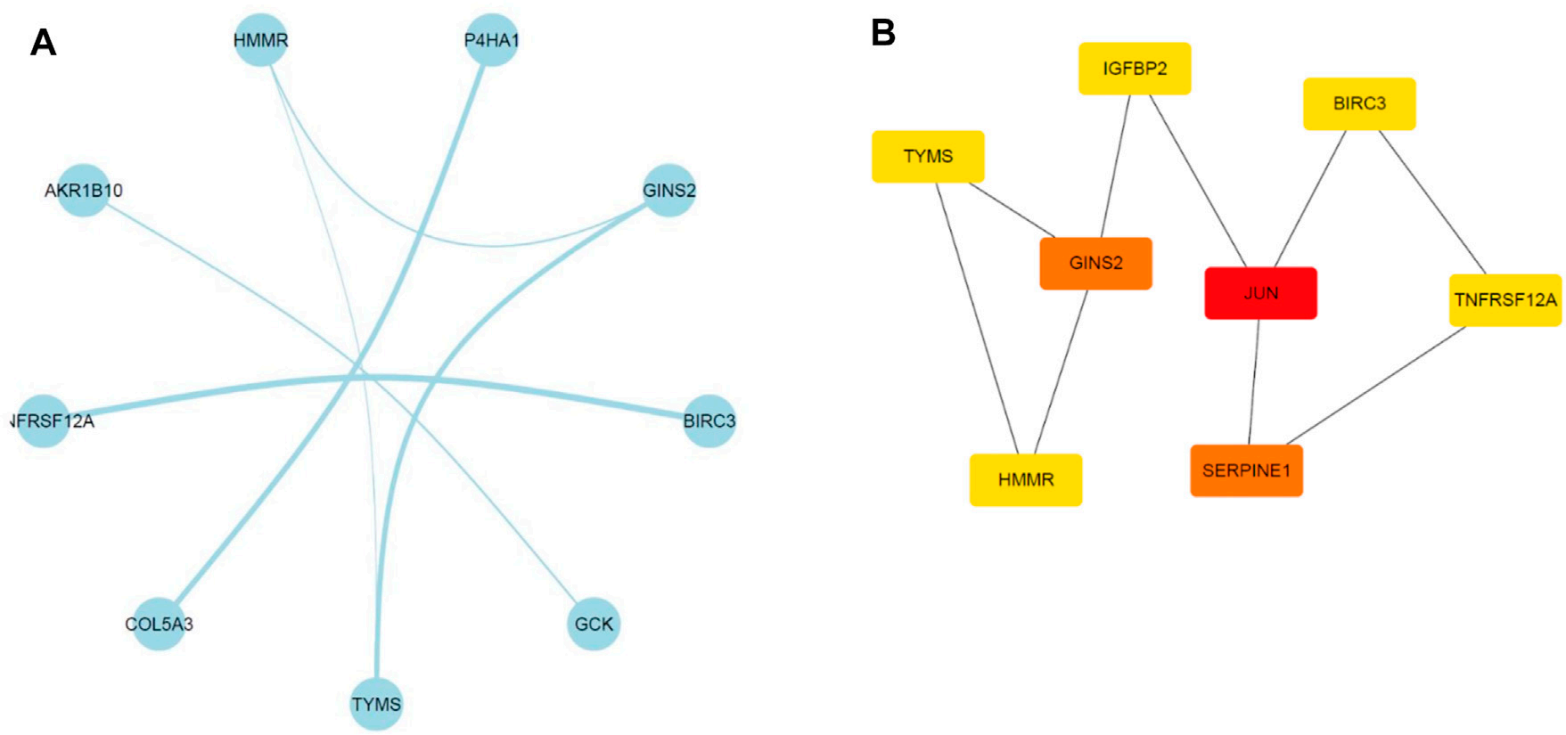
Illustration of the PPI networks. **(A)** PPI network of the genes between DEG lists and co-expression modules. The nodes represent the genes. Edges indicate interaction associations between nodes. **(B)** Identification of the hub genes through maximal clique centrality (MCC) algorithm. Edges represent the protein-protein associations. The red nodes represent genes with a high MCC sore, and the yellow ones represent genes with a low MCC sore.

A PPI network involving the overlapping genes was constructed (Figure 7A). Based on the MCC score, the top 8 highest-scored genes, including JUN, SERPINE1, GINS2, TYMS, HMMR, IGFBP2, BIRC3 and TNFRSF12A, were finally identified as hub genes. The gene symbols, full names and MCC scores were displayed in Table 3. Figure 7B illustrates the PPI network of the 8 hub genes.

**TABLE 3:** Top 8 hub genes based on MCC score.

Gene Symbol	Full Name	MCC Score
JUN	Jun proto-oncogene	4
SERPINE1	serpin family E member 1	3
GINS2	GINS complex subunit 2	3
TYMS	thymidylate synthetase	2
HMMR	hyaluronan mediated motility receptor	2
IGFBP2	insulin like growth factor binding protein 2	2
BIRC3	baculoviral IAP repeat containing 3	2
TNFRSF12A	TNF receptor superfamily member 12A	2

### Validation of the Hub Genes Through a GEO Dataset

In the present study, dataset GSE164760 was used for verification of the expression patterns of hub genes. In comparison to normal liver tissues, three (JUN, SERPINE1, IGFBP2) of the eight hub genes were shown to be significantly downregulated in NASH (Table 4).

**TABLE 4:** Expression pattern of potential hub genes in dataset GSE164760.

Gene Symbol	logFC (NASH *vs*. HC)	Adjusted *p*-Value
JUN	−1.17	3.23E-04
SERPINE1	−1.08	1.06E-02
GINS2	−0.11	6.52E-01
TYMS	0.07	7.61E-01
HMMR	0.12	3.47E-01
IGFBP2	−1.29	4.37E-01
BIRC3	0.01	9.78E-01
TNFRSF12A	−0.60	2.16E-02

### Validation of the Expression Levels of Hub Genes *in Vivo*

**FIGURE 8:**
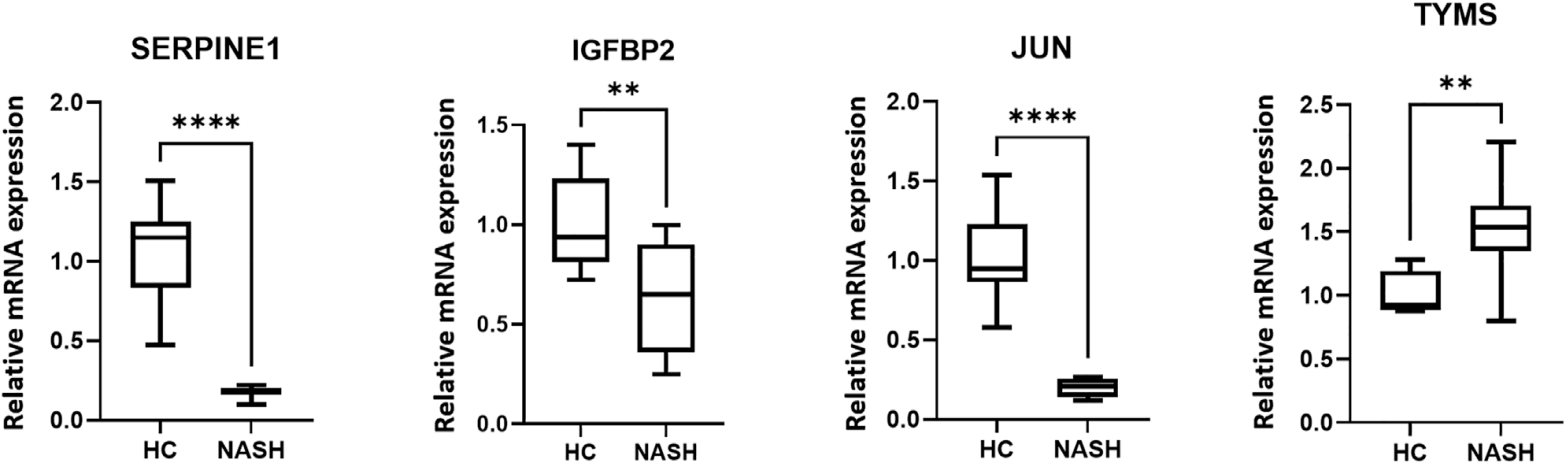
Gene expression in a mouse model of NASH and in HC. ****p < 0.0001, **p < 0.01.

In the animal experiment, we found that all the three verified genes (JUN, SERPINE1, IGFBP2) were significantly downregulated in the HFD/CCl4 treated group compared to the HC group. The relative mRNA expression of the three genes were shown in Figure 8.

## Discussion

Despite the fact that NASH has become a serious public health problem worldwide, the exact mechanism behind it still remains unclear. Moreover, while liver biopsy is the gold standard for the diagnosis of NASH, it is an intrusive operation with risks and drawbacks. Therefore, developing biomarkers for noninvasive diagnosis of NASH is a pressing need (Castera et al., 2019).

Recently, studies have emerged using bioinformatic tools to explore the mechanisms underlying diseases within the metabolic syndrome. One of the most frequently used analysis methods is DEG analysis. Several studies have identified key genes in dietary end products-induced NAFLD (Wang et al., 2020) or fulminant type 1 diabetes based on this method (Ye et al., 2020). Each of them dealt with a specific aspect and provided deeper understanding of the diseases. WGCNA was also used to analyze gene expression patterns, and it was frequently used in conjunction with DEG analysis. Wu et al. conducted WGCNA on two datasets from GEO and identified hub genes of NAFLD (Wu et al., 2021). Zeng et al. used similar methods, but a different dataset to find the hub genes in NAFLD (Zeng et al., 2021). Other bioinformatic tools, such as immune infiltration analysis, were also used in combination with DEG to explore the molecular mechanisms of NASH (Jiang et al., 2021). The key genes found in these studies require further preclinical and clinical investigation.

In this study, 24 differential co-expression genes were found in the GSE89632 and GSE126848 datasets using integrated bioinformatic analysis. According to GO analysis, these genes were mainly enriched in circadian rhythm, aging, angiogenesis, response to drug, endoplasmic reticulum lumen and protein binding. KEGG analysis demonstrated that they were mostly enriched in pathways of focal adhesion and galactose metabolism. Based on MCC score, the top 8 genes were designated as hub genes.

Among the eight hub genes, three (JUN, SERPINE1, IGFBP2) were found to be significantly downregulated in NASH compared with HC using a validation dataset. We also found that these three genes were expressed significantly at lower levels in the HFD/CCl4 induced mouse model of NASH than in normal controls.

JUN, also known as c-Jun, is a proto-oncogene. Findings in hepatocytes have implicated that the reduced glutathione level due to oxidative stress leads to overactivation of c-Jun N-terminal kinase (JNK)/c-Jun signaling that induces cell death (Wang et al., 2010). However, in our study, the mRNA level of JUN was downregulated in the validation dataset and the animal model of NASH, which is probably because the activation of the signaling pathway is not necessarily accomplished by mRNA overexpression.

SERPINE1, also known as PAI-1, is a member of the serine proteinase inhibitor (serpin) superfamily that plays a profibrotic role in a variety of organs (Ghosh and Vaughan, 2012). According to prior research, patients with NAFLD have higher plasma and hepatic levels of PAI-1 (Sookoian et al., 2010). It was found in clinical cohorts that the degree of rise in blood PAI-1 levels has been linked to the degree of liver steatosis (Barbato et al., 2009), inflammation, and fibrosis (Verrijken et al., 2014). These findings suggest that PAI-1 may be involved in the mechanisms of NASH-related fibrosis, while the underlying processes are still unknown. In a prior study, an oral PAI-1 inhibitor was utilized to treat mice fed a high-fat diet. Hepatic steatosis was reduced in both early and delayed treatments, but only the early therapy prevented inflammation and fibrosis (Lee et al., 2017). In another study in mice, the inhibition of SERPINE1 reduced hepatic steatosis but not inflammation or fibrosis. These results suggest that SERPINE1 is likely to control hepatic lipid accumulation rather than drive NASH development (Henkel et al., 2018). However, in this study, hepatic SERPINE1 levels were downregulated in NASH, which was validated by a human dataset and a mouse model. On one hand, this may be a bias due to the small sample size. On the other hand, because of the uncertainty of the underlying mechanisms, this finding suggests that the exact role of SERPINE1 requires further investigation.

IGFBP2 is a gene that codes for one of the six proteins that bind insulin like growth factors 1 and 2 (IGF1 and IGF2). Hepatic glucose and lipid metabolism are regulated by growth factor and IGF1, and their downregulation may contribute to NAFLD (Stanley et al., 2021). Recent studies in human showed that NALFD is correlated with lower hepatic mRNA level of IGFBP2 (Dali-Youcef et al., 2019; Stanley et al., 2021). What’s more, hepatic IGFBP2 expression correlates with its circulating level and is associated with hepatic metabolism and lipogenesis. Xu et al. reported that overexpression of IGFBP2 blunted the accumulation of triglycerides in HepG2 cells (Chen et al., 2021). Pia et al. showed that IGFBP2 attenuated lipogenesis stimulated by IGF1 in hepatocytes (Fahlbusch et al., 2020). In this study, we also found that hepatic IGFBP2 levels decreased in NASH in all of the three human mRNA datasets as well as in the animal model, suggesting the potential of IGFBP2 to be a noninvasive biomarker for the diagnosis of NASH.

Additionally, it should be noted that TYMS was significantly upregulated in GSE89632 ( $\log_{2}FC$ = 1.56) and GSE126848 ( $\log_{2}FC$ = 2.02) and the in mouse model of NASH (Figure 8). This gene encodes thymidylate synthetase, which acts as a critical enzyme in the *de novo* dTMP synthesis, and plays a vital role in maintaining mitochondrial functions (Anderson et al., 2011). Previous studies have indicated that impaired mitochondrial function is a potential mechanism of liver damage in NAFLD patients (Tarantino et al., 2014). However, the levels of TYMS were upregulated in our study, which means it may not damage the liver through the mitochondrial dysfunction mechanism.

Despite the fact that our study enhances the study of NASH mechanisms and provides possible biomarkers for noninvasive NASH diagnosis, it has the following limitations: (Massoud and Charlton, 2018). It's still unclear whether the inferred alterations in expression are causes or consequences of the disease (Younossi, 2019). The sample size of this study was relatively small, which weakened the reliability of the conclusions. Therefore, we will seek to collect more clinical samples of NASH patients to further verify the findings of this study (Chalasani et al., 2018). In the future, the underlying molecular mechanisms that reveal the exact roles of the hub genes should be studied more thoroughly by *in vivo* and *in vitro* experiments.

## Data Availability

The datasets presented in this study can be found in online repositories. The names of the repository/repositories and accession number(s) can be found in the article/Supplementary Material.

## Abbreviations

BP, biological process; CC, cellular component; CCl4, carbon tetrachloride; DEG, differentially expressed genes; FC, fold change; FDR, false discovery rate; GEO, Gene Expression Omnibus; GO, Gene Ontology; HC, healthy controls; HCC, hepatocellular carcinoma; HFD, high-fat diet; IGF1: insulin like growth factor 1; IGF2: insulin like growth factor 2; IGFBP2, insulin like growth factor binding protein 2; JNK, c-Jun N-terminal kinase; JUN, Jun proto-oncogene; KEGG, Kyoto Encyclopaedia of Genes and Genomes; MCC, Maximal Clique Centrality; MF, molecular function; NAFLD, non-alcoholic fatty liver disease; NASH, non-alcoholic steatohepatitis; NAFL, non-alcoholic fatty liver; PPI, protein-protein interaction; SERPINE1, serpin family E member 1; STRING, Search Tool for the Retrieval of Interacting Genes; TOM, topological overlap matrix; WGCNA, weighted gene co-expression network analysis.

## References

[B1] Anderson D. D. Quintero C. M. Stover P. J. (2011). Identification of a De Novo Thymidylate Biosynthesis Pathway in Mammalian Mitochondria. Proc. Natl. Acad. Sci. United States America 108 (37), 15163–15168. 10.1073/pnas.1103623108 PMC3174652 21876188

[B2] Barbato A. Iacone R. Tarantino G. Russo O. Sorrentino P. Avallone S. (2009). Relationships of PAI-1 Levels to central Obesity and Liver Steatosis in a Sample of Adult Male Population in Southern Italy. Intern. Emerg. Med. 4 (4), 315–323. 10.1007/s11739-009-0240-9 19350365

[B3] Can T. (2014). Introduction to Bioinformatics. Methods Mol. Biol. (Clifton, NJ) 1107, 51–71. 10.1007/978-1-62703-748-8_4 24272431

[B4] Castera L. Friedrich-Rust M. Loomba R. (2019). Noninvasive Assessment of Liver Disease in Patients with Nonalcoholic Fatty Liver Disease. Gastroenterology 156 (5), 1264–1281. 10.1053/j.gastro.2018.12.036 30660725 PMC7505052

[B5] Chalasani N. Younossi Z. Lavine J. E. Charlton M. Cusi K. Rinella M. (2018). The Diagnosis and Management of Nonalcoholic Fatty Liver Disease: Practice Guidance from the American Association for the Study of Liver Diseases. Hepatology 67 (1), 328–357. 10.1002/hep.29367 28714183

[B6] Chen H. Boutros P. C. (2011). VennDiagram: a Package for the Generation of Highly-Customizable Venn and Euler Diagrams in R. BMC bioinformatics 12, 35. 10.1186/1471-2105-12-35 21269502 PMC3041657

[B7] Chen X. Tang Y. Chen S. Ling W. Wang Q. (2021). IGFBP-2 as a Biomarker in NAFLD Improves Hepatic Steatosis: an Integrated Bioinformatics and Experimental Study. Endocr. connections 10 (10), 1315–1325. 10.1530/ec-21-0353 PMC8562889 34524971

[B8] Chin C-H. Chen S-H. Wu H-H. Ho C-W. Ko M-T. Lin C-Y. (2014). cytoHubba: Identifying Hub Objects and Sub-networks from Complex Interactome. BMC Syst. Biol. 8 (Suppl. 4:S11). 10.1186/1752-0509-8-S4-S11 PMC4290687 25521941

[B9] Dali-Youcef N. Vix M. Costantino F. El-Saghire H. Lhermitte B. Callari C. (2019). Interleukin-32 Contributes to Human Nonalcoholic Fatty Liver Disease and Insulin Resistance. Hepatol. Commun. 3 (9), 1205–1220. 10.1002/hep4.1396 31497742 PMC6719754

[B10] Fahlbusch P. Knebel B. Hörbelt T. Barbosa D. M. Nikolic A. Jacob S. (2020). Physiological Disturbance in Fatty Liver Energy Metabolism Converges on IGFBP2 Abundance and Regulation in Mice and Men. Int. J. Mol. Sci. 21 (11). 10.3390/ijms21114144 PMC7312731 32532003

[B11] Franceschini A. Szklarczyk D. Frankild S. Kuhn M. Simonovic M. Roth A. (2013). STRING v9.1: Protein-Protein Interaction Networks, with Increased Coverage and Integration. Nucleic Acids Res. 41 (Database issue), D808–D15. 10.1093/nar/gks1094 23203871 PMC3531103

[B12] Friedman S. L. Neuschwander-Tetri B. A. Rinella M. Sanyal A. J. (2018). Mechanisms of NAFLD Development and Therapeutic Strategies. Nat. Med. 24 (7), 908–922. 10.1038/s41591-018-0104-9 29967350 PMC6553468

[B13] Ghosh A. K. Vaughan D. E. (2012). PAI-1 in Tissue Fibrosis. J. Cell. Physiol. 227 (2), 493–507. 10.1002/jcp.22783 21465481 PMC3204398

[B14] Henkel A. S. Khan S. S. Olivares S. Miyata T. Vaughan D. E. (2018). Inhibition of Plasminogen Activator Inhibitor 1 Attenuates Hepatic Steatosis but Does Not Prevent Progressive Nonalcoholic Steatohepatitis in Mice. Hepatol. Commun. 2 (12), 1479–1492. 10.1002/hep4.1259 30556037 PMC6287480

[B15] Ito K. Murphy D. (2013). Application of Ggplot2 to Pharmacometric Graphics. CPT Pharmacometrics Syst. Pharmacol. 2, e79. 10.1038/psp.2013.56 24132163 PMC3817376

[B16] Jiang Z. Y. Zhou Y. Zhou L. Li S. W. Wang B. M. (2021). Identification of Key Genes and Immune Infiltrate in Nonalcoholic Steatohepatitis: A Bioinformatic Analysis. Biomed. Research International 2021, 7561645. 10.1155/2021/7561645 34552988 PMC8452393

[B17] Kubota N. Kado S. Kano M. Masuoka N. Nagata Y. Kobayashi T. (2013). A High-Fat Diet and Multiple Administration of Carbon Tetrachloride Induces Liver Injury and Pathological Features Associated with Non-alcoholic Steatohepatitis in Mice. Clin. Exp. Pharmacol. Physiol. 40 (7), 422–430. 10.1111/1440-1681.12102 23611112

[B18] Langfelder P. Horvath S. (2008). WGCNA: an R Package for Weighted Correlation Network Analysis. BMC bioinformatics 9, 559. 10.1186/1471-2105-9-559 19114008 PMC2631488

[B19] Lee S. M. Dorotea D. Jung I. Nakabayashi T. Miyata T. Ha H. (2017). TM5441, a Plasminogen Activator Inhibitor-1 Inhibitor, Protects against High Fat Diet-Induced Non-alcoholic Fatty Liver Disease. Oncotarget 8 (52), 89746–89760. 10.18632/oncotarget.21120 29163785 PMC5685706

[B20] Massoud O. Charlton M. (2018). Nonalcoholic Fatty Liver Disease/Nonalcoholic Steatohepatitis and Hepatocellular Carcinoma. Clin. Liver Dis. 22 (1), 201–211. 10.1016/j.cld.2017.08.014 29128057

[B21] Ritchie M. E. Phipson B. Wu D. Hu Y. Law C. W. Shi W. (2015). Limma powers Differential Expression Analyses for RNA-Sequencing and Microarray Studies. Nucleic Acids Res. 43 (7), e47. 10.1093/nar/gkv007 25605792 PMC4402510

[B22] Sanyal A. J. (2019). Past, Present and Future Perspectives in Nonalcoholic Fatty Liver Disease. Nat. Rev. Gastroenterol. Hepatol. 16 (6), 377–386. 10.1038/s41575-019-0144-8 31024089

[B23] Saris C. G. Horvath S. van Vught P. W. van Es M. A. Blauw H. M. Fuller T. F. (2009). Weighted Gene Co-expression Network Analysis of the Peripheral Blood from Amyotrophic Lateral Sclerosis Patients. BMC genomics 10, 405. 10.1186/1471-2164-10-405 19712483 PMC2743717

[B24] Segundo-Val I. S. Sanz-Lozano C. S. (2016). Introduction to the Gene Expression Analysis, Methods Mol. Biol., 1434, 29–43. 10.1007/978-1-4939-3652-6_3 27300529

[B25] Sookoian S. Castaño G. O. Burgueño A. L. Rosselli M. S. Gianotti T. F. Mallardi P. (2010). Circulating Levels and Hepatic Expression of Molecular Mediators of Atherosclerosis in Nonalcoholic Fatty Liver Disease. Atherosclerosis 209 (2), 585–591. 10.1016/j.atherosclerosis.2009.10.011 19896127

[B26] Stanley T. L. Fourman L. T. Zheng I. McClure C. M. Feldpausch M. N. Torriani M. (2021). Relationship of IGF-1 and IGF-Binding Proteins to Disease Severity and Glycemia in Nonalcoholic Fatty Liver Disease. J. Clin. Endocrinol. Metab. 106 (2), e520–e33. 10.1210/clinem/dgaa792 33125080 PMC7823253

[B27] Sumida Y. Nakajima A. Itoh Y. (2014). Limitations of Liver Biopsy and Non-invasive Diagnostic Tests for the Diagnosis of Nonalcoholic Fatty Liver Disease/nonalcoholic Steatohepatitis. World J. Gastroenterol. 20 (2), 475–485. 10.3748/wjg.v20.i2.475 24574716 PMC3923022

[B28] Tarantino G. Finelli C. Scopacasa F. Pasanisi F. Contaldo F. Capone D. (2014). Circulating Levels of Sirtuin 4, a Potential Marker of Oxidative Metabolism, Related to Coronary Artery Disease in Obese Patients Suffering from NAFLD, with normal or Slightly Increased Liver Enzymes. Oxidative Med. Cell. longevity 2014, 920676. 10.1155/2014/920676 PMC4086623 25045415

[B29] Verrijken A. Francque S. Mertens I. Prawitt J. Caron S. Hubens G. (2014). Prothrombotic Factors in Histologically Proven Nonalcoholic Fatty Liver Disease and Nonalcoholic Steatohepatitis. Hepatology 59 (1), 121–129. 10.1002/hep.26510 24375485

[B30] Wang J. Liu H. Xie G. Cai W. Xu J. (2020). Identification of Hub Genes and Key Pathways of Dietary Advanced Glycation End Products-Induced Non-alcoholic Fatty Liver Disease by Bioinformatics Analysis and Animal Experiments. Mol. Med. Rep. 21 (2), 685–694. 10.3892/mmr.2019 31974594 PMC6947946

[B31] Wang Y. Singh R. Xiang Y. Czaja M. J. (2010). Macroautophagy and Chaperone-Mediated Autophagy Are Required for Hepatocyte Resistance to Oxidant Stress. Hepatology (Baltimore, Md) 52 (1), 266–277. 10.1002/hep.23645 PMC2924621 20578144

[B32] Wu C. Zhou Y. Wang M. Dai G. Liu X. Lai L. (2021). Bioinformatics Analysis Explores Potential Hub Genes in Nonalcoholic Fatty Liver Disease. Front. Genet. 12, 772487. 10.3389/fgene.2021.772487 34777484 PMC8586215

[B33] Yang Y. Han L. Yuan Y. Li J. Hei N. Liang H. (2014). Gene Co-expression Network Analysis Reveals Common System-Level Properties of Prognostic Genes across Cancer Types. Nat. Commun. 5, 3231. 10.1038/ncomms4231 24488081 PMC3951205

[B34] Ye X. Zeng T. Kong W. Chen L. L. (2020). Integrative Analyses of Genes Associated with Fulminant Type 1 Diabetes. J. Immunol. Res. 2020, 1025857. 10.1155/2020/1025857 33083497 PMC7559223

[B35] Younossi Z. M. (2019). Non-alcoholic Fatty Liver Disease - A Global Public Health Perspective. J. Hepatol. 70 (3), 531–544. 10.1016/j.jhep.2018.10.033 30414863

[B36] Yu G. Wang L-G. Han Y. He Q-Y. (2012). clusterProfiler: an R Package for Comparing Biological Themes Among Gene Clusters. OMICS 16 (5), 284–287. 10.1089/omi.2011.0118 22455463 PMC3339379

[B37] Zeng F. Shi M. Xiao H. Chi X. (2021). WGCNA-based Identification of Hub Genes and Key Pathways Involved in Nonalcoholic Fatty Liver Disease. Biomed. Research International 2021, 5633211. 10.1155/2021/5633211 34938809 PMC8687832

[B38] Zhang B. Horvath S. (2005). A General Framework for Weighted Gene Co-expression Network Analysis. Stat. Appl. Genet. Mol. Biol. 4, Article17. 10.2202/1544-6115.1128 16646834

